# A positive contribution to nitrogen removal by a novel NOB in a full-scale duck wastewater treatment system

**DOI:** 10.1016/j.wroa.2024.100237

**Published:** 2024-07-10

**Authors:** Pengfei Hu, Youfen Qian, Yanbin Xu, Adi Radian, Yuchun Yang, Ji-Dong Gu

**Affiliations:** a Civil and Environmental Engineering, Technion – Israel Institute of Technology, Haifa 320003, Israel; b Environmental Science and Engineering Research Group, Guangdong Technion - Israel Institute of Technology, 241 Daxue Road, Shantou, Guangdong 515063, People’s Republic of China; c School of Environmental Sciences and Engineering, Guangdong University of Technology, Guangzhou, Guangdong 510006, People’s Republic of China; d State Key Laboratory of Biocontrol, School of Ecology, Sun Yat-sen University, Guangzhou, Guangdong 510275, People’s Republic of China; e Guangdong Provincial Key Laboratory of Materials and Technologies for Energy Conversion, Guangdong Technion - Israel Institute of Technology, 241 Daxue Road, Shantou, Guangdong 515063, People’s Republic of China

**Keywords:** Ammonia oxidation, Nitrite oxidation, Nitrogen removal, Wastewater treatment

## Abstract

Nitrite-oxidizing bacteria (NOB) are undesirable in the anaerobic ammonium oxidation (anammox)-driven nitrogen removal technologies in the modern wastewater treatment plants (WWTPs). Diverse strategies have been developed to suppress NOB based on their physiological properties that we have understood. But our knowledge of the diversity and mechanisms employed by NOB for survival in the modern WWTPs remains limited. Here, Three NOB species (NOB01–03) were recovered from the metagenomic datasets of a full-scale WWTP treating duck breeding wastewater. Among them, NOB01 and NOB02 were classified as newly identified lineage VII, tentatively named *Candidatus* (*Ca.*) Nitrospira NOB01 and *Ca.* Nitrospira NOB02. Analyses of genomes and in situ transcriptomes revealed that these two novel NOB were active and showed a high metabolic versatility. The transcriptional activity of *Ca.* Nitrospira could be detected in all tanks with quite different dissolved oxygen (DO) (0.01–5.01 mg/L), illustrating *Ca.* Nitrospira can survive in fluctuating DO conditions. The much lower *Ca.* Nitrospira abundance on the anammox bacteria-enriched sponge carrier likely originated from the intensification substrate (NO_2_^−^) competition from anammox and denitrifying bacteria. In particular, a highlight is that *Ca.* Nitrospira encoded and treanscribed cyanate hydratase (CynS), amine oxidase, urease (UreC), and copper-containing nitrite reductase (NirK) related to ammonium and NO production, driving NOB to interact with the co-existed AOB and anammox bacteria. *Ca.* Nitrospira strains NOB01 and NOB02 showed quite different niche preference in the same aerobic tank, which dominanted the NOB communities in activated sludge and biofilm, respectively. In addition to the common rTCA cycle for CO_2_ fixation, a reductive glycine pathway (RGP) was encoded and transcribed by NOB02 likely for CO_2_ fixation purpose. Additionally, a 3b group hydrogenase and respiratory nitrate reductase were uniquely encoded and transcribed by NOB02, which likely confer a survival advantage to this strain in the fluctuant activated sludge niche. The discovery of this new genus significantly broadens our understanding of the ecophysiology of NOB. Furthermore, the impressive metabolic versatility of the novel NOB revealed in this study advances our understanding of the survival strategy of NOB and provides valuable insight for suppressing NOB in the anammox-based WWTP.

## Highlights

• Three new nitrite-oxidizing bacteria (NOB) were recovered from full-scale WWTP.
• Two of the NOBs were active showing high metabolic versatility in sludge and biofilms co-metabolizing with others.
• A reductive glycine pathway (RGP) was transcribed by NOB02 likely for CO2 fixation.
• Functional determination of the *nitraspira* and *Ca.* Nitrospira for potential gene transfer.

## Graphical abstract

**Figure:**
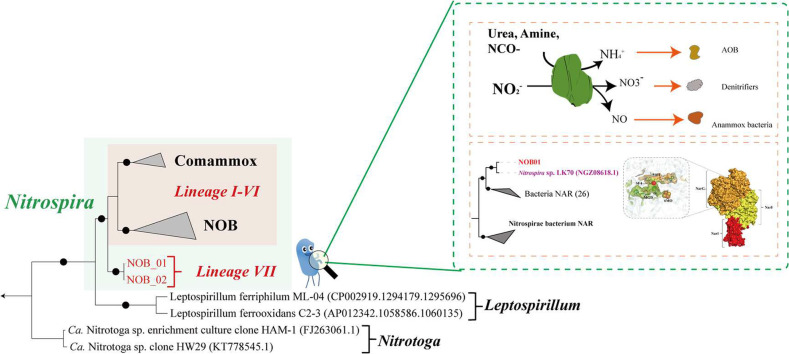

## Introduction

Ammonia (NH_3_) oxidation, also named nitrification, is an essential process in various ecosystems, including marine and terrestrial environments for ammonia balance (Kuypers et al., 2018). Nitrification is driven by autotrophic microorganisms that are growing with NH_3_ or nitrite (NO_2_^−^) as energy-generating substrates. The nitrification process has long been believed to be carried out by two different communities, the conversion of ammonia to NO_2_^−^ by autotrophic ammonia-oxidizing bacteria (AOB) and archaea (AOA) (Kuypers et al. 2018; Santoro et al. 2015), and the oxidation of NO_2_^−^to NO_3_^−^ by chemolithoautotrophic nitrite-oxidizing bacteria (NOB). NOB were considered phylogenetically heterogeneous nitrifiers and widespread distribution in nature and man-made ecosystems (Bayer et al. 2021; Lücker et al. 2010, Leung et al. 2022). To date, characterized NOB are mainly affiliated with twelve genera, including *Nitrobacter, Nitrococcus, Nitrospina, Nitrospira, Nitrospirota, Nitrotoga, Nitrolancea, Candidatus* (*Ca.*) Nitromaritima, *Ca.* Nitrocaldera*, Ca.* Nitrotheca*, Ca.* Nitrohelix *and Ca.* Nitronauta (Daims et al. 2016; Daims and Wagner 2018; Su et al. 2023), suggesting an extensive phylogenetic pedigree of NOB. Comparatively, NOB *Nitrospira* in the family Nitrospiraceae is the most phylogenetically diverse and abundant NOB in various ecosystems, such as soil, oceans, freshwater, hot spring, and wastewater (Bayer et al. 2021; Daims et al. 2016; Liu et al. 2021; Ushiki et al. 2017b). Based on the phylogenetic analysis of the 16S rRNA gene sequence, *Nitrospira* was assigned to seven different phylogenetic lineages, in which lineages I, II and VII have been ubiquitously identified in wastewater treatment plants (WWTPs) (Gruber-Dorninger et al. 2015; Keuter et al. 2023; Ushiki et al. 2017a). Also, another NOB that is widely known is *Nitrobacter*, which belongs to the *Alphaproteobacteria*. Unlike *Nitrospira*, which prefers to survive in nitrite-limited and oxygen-minimum zones, resulting from the *K*-strategists with high affinity for nitrite and oxygen, NOB *Nitrobacter* is an *r*-strategists, that could tolerant high substrate concentrate (Nowka et al. 2015). Additionally, a recent study also indicated that *Nitrospira* moscoviensis could survive in nitrite-limited conditions because the presence of 2a [NiFe]-hydrogenase enabled it for aerobic growth using H_2_ as the sole energy source (Leung et al. 2022), and benefit from the mixotrophic lifestyle, including pathways for the transport, oxidation, and assimilation of simple organic compounds and the reverse tricarboxylic acid cycle, *Nitrospira* defluvii can survive in substrate-limited conditions (Lücker et al. 2010). Considering that the phylogenetic tree of functional classification has been constantly developed due to the expansion of available genomic data, such as bacteria involved in H_2_ oxidation (Koch et al. 2015, Leung et al. 2022), various carbon fixation pathways (Lücker et al. 2010; Starkenburg et al. 2006) and ammonia oxidation (Daims et al. 2015; van Kessel et al. 2015). An open question is still unclear whether the currently characterized NOB lineages can fully represent the diversity and metabolism of NOB. To better explain this question, it is imperative to recover more distinct branching lineages of NOB that will advance the elucidation of ecophysiological extension and evolutionary history.

A stable supply of nitrite is a prerequisite for the anammox-based nitrogen removal technologies, one of the major issue is to control the nitrite oxidation activity of NOB in WWTPs. AOB could survive in an extremely low DO condition (0.2–0.4 mg/L), which was much lower than that for NOB (1.5–12 mg/L) (Picioreanu et al. 1997). This provides a new inspiration that a low DO environment favors the growth of AOB and inhibits the activity of NOB. Whereas, following studies found that the oxygen affinity of various NOB is quite different. For example, *Nitrospira*-like nitrite oxidizer could survive in a much low DO concentration of 0.5 mg/L, a high DO concentration facilitated the growth of *Nitrobacter* (Daims et al. 2001; Regmi et al. 2014). It was suggested that, in order to achieve NOB suppression, the DO concentration should be controlled at a moderate level of 1.0 mg/L or a lower level of 0.5 mg/L (Cao et al. 2017). In addition, a previous study also indicated that NOB have a longer lag time than AOB during the transition from anoxic to oxic conditions due to the deactivation of hydroxylamine (Xu et al. 2012). As such, an intermittent aeration-based operation strategy had been proposed for suppressing NOB activity by creating a transient anoxic condition in SBR or continuous step-feed reactors (Gu et al. 2018; Miao et al. 2018). Most recently, a novel process of partial nitrification, anammox, and methane-dependent nitrite/nitrate reduction (PNAM) has been developed and applied in a single membrane biofilm reactor (MBfR) (Liu et al. 2021). In this system, NOB competes for nitrite with anammox bacteria, nitrite/nitrate-dependent anaerobic methane oxidation (n-DAMO) bacteria, and archaea, and thereby NOB can be maintained at a lower abundance by controlling the input of methane (Liu et al. 2021). This process, however, also encounters an unavoidable problem with the harsh oxygen availability, which directly affects the ammonia oxidation and overall nitrogen removal efficiencies. Above strategies have obtained a certain success in NOB suppression mostly in laboratorial bioreactors, but there are still many challenges for practical application in full-scale WWTPs. One of the crucial reasons is the extensive phylogenetic pedigree of NOB in WWTP with quite different physiologic properties, but our understanding on the ecophysiology of NOB remains limited. Therefore, more efforts are still needed to reveal the diversity and metabolic versatility of NOB in wastewater treatment systems, which will provide variluable insights on optimal WWTP configurations by suppressing NOB for efficient anammox-based nitrogen removal.

Here, metagenome and metatranscriptome analyses were performed on four distinct niches of a full-scale WWTP treating duck breeding wastewater, including two anaerobic tanks, one anoxic tank and one oxic tank. Two high-quality metagenome-assembled genomes (MAGs) representing a new NOB lineage were retrieved from the WWTP. These two novel NOB dominated the NOB communities in different reactors of the WWTP, but with the lowest abundances and transcriptional activities in the anoxic tank, reflecting the growth of NOB was significantly inhibited. By introducing the genomic characterization and transcriptional results, we revealed a high metabolic versatility and survival advantage of the novel NOB in wastewater treatment systems. Environmental conditions that inhibited the growth of NOB in the anoxic tank were further investigated.

## Results and discussion

### Recovery of novel NOB species from the full-scale WWTP

After shotgun sequencing and binning analysis, we retrieved a total of 27 NOB metagenome-assembled genomes (MAGs) from different reactors of the WWTP (Table S1). Based on the average nucleotide identity (ANI) and 43 concatenated markers phylogenetic analysis, the 27 MAGs were divided into four groups (Fig. S1, Table S1). After homologous gene prediction and comparison, 99.8–100 % identity was shared by MAGs within each group. So that four representative high-quality (Completeness > 91 %, Contamination < 7 %) draft genome assemblies (NOB01, NOB02, NOB03, and NOB04) from these four groups were selected for downstream analysis.

**Fig. 1:**
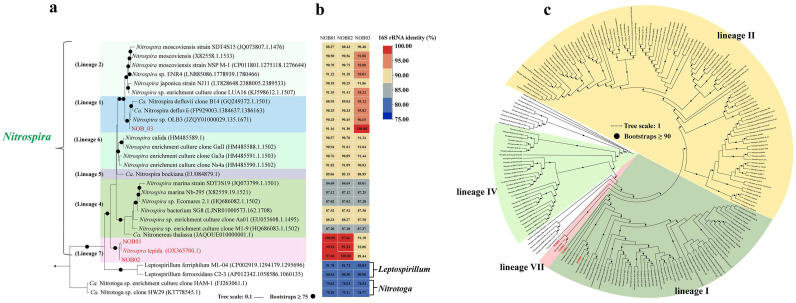
Phylogenetic analyses of 16S rRNA gene (a) and identity (b) show the affiliations of these three new NOB bacteria (red font) and known NOB strains (black font), **Maximum likelihood tree of 43 concatenated markers gene (c)** show the affiliations of retained for NOB bacteria (red font) and known NOB strains (black font). 16S rRNA gene tree was rooted by 16S rRNA genes of *Nitrotoga*, 43 markers phylogenetic tree was rooted by midpoint.

The preliminary classification carried out by the GTDB-tk could assigned NOB01, NOB02, NOB03 and NOB04 were classified as genus *Nitrospira* (Fig. S4). Consistently, phylogenetic analysis based on the 43 concatenated markers indicates that NOB03 and NOB04 had the closest lineage with “*Nitrospira* defluvii NOB2” (Fig. 1b). The highest ANI between NOB04 and the known NOB was 99.8 % (Table S2), larger than the recommended species cutoff (ANI > 95 %) (Konstantinidis et al. 2017), we thus named it “*Ca*. Nitrospira defluvii NOB04”. The highest ANI between NOB03 and the known NOB was 74.98 % (Fig. 1b, Table S2), lower than 95 %, but it shared a 98.81 % 16S rRNA gene identity with “*Ca.* Nitrospira sp. OLB3”, larger than the proposed species cutoff based on 16S rRNA gene (> 98.6%). Thereby, NOB03 was considered as a same species with “*Ca.* Nitrospira sp. OLB3” in the *Nitrospira* genus and named it “*Ca.* Nitrospira sp. NOB03”. Interestingly, the maximum ANI of NOB01 with the known *Nitrospira* genomes were 85.33 % than the recommended species cutoff (ANI > 95 %), while NOB02 share 99.90 % ANI with *Nitrospira* tebida (Table S2) (Keuter et al. 2023). Similarly, nearly full-length 16S rRNA gene sequences were successfully extracted from MAGs NOB01, NOB02 and a maximum likelihood tree based on 16S rRNA gene sequences was constructed (Fig. 1a). Comparable with the result of phylogenetic analysis based on the 43 concatenated markers, NOB01 and NOB02 formed a separate cluster with *Nitrospira* tebida (Fig. 1c). The nearly full-length 16S rRNA gene sequences of NOB01 and NOB02 were most similar (99.93 % and 99.33 %, respectively) to the 16S rRNA gene from *Nitrospira* tebida, which is larger than the genus cutoff based on 16S rRNA gene (94.5 %) (Yarza et al. 2014). Therefore, we proposed NOB01 and NOB02 belonge to the novel lineage VII, which were named as “*Ca*. Nitrospira NOB01” and “*Ca*. Nitrospira sp. NOB02”.

### Active nitrogen metabolism of two lineage VII members in WWTP

**Fig. 2:**
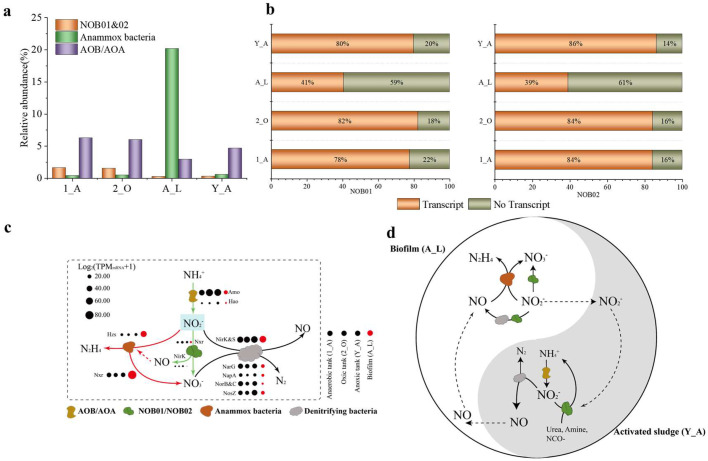
**NOB *Nitrospirae* actively participated in nitrogen cycle in the full-scale WWTP with close cooperation with other organisms. (a) Relative abundances of nitrogen cycle related microorganisms in different niches.** Relative abundance = TPM/total TPM *100 %. **(b) Percentages of the *in-situ* transcribed genes of the two novel NOB. (c) The overall nitrogen metabolism networks in the four niches with the participation of the two novel NOB. (d) The constructed co-metabolism model between activated sludge (Y_A) and biofilm (A_L) niches in the anoxic tank (1_A) carried out by nitrogen cycle related microorganisms.** Amo, ammonia oxidoreductase; Hao, Hydroxylamine oxidoreductase; Nxr, nitrite oxidoreductase; NirK, nitrite reductase (Cu-forming); NapA, periplasmic dissimilatory nitrate reductases; Nor, nitric oxide reductase; NosZ, nitrous-oxide reductase. Anaerobic tank (1_A), Anoxic tank (1_O), Anaerobic tank (2_A) and Oxic tank (2_O). Y_A and Y_B represent two separated parts in Anoxic tank (1_O).

Around 80 % of the genes in both NOB01 and NOB02 in activated sludge niches (1_A, 2_A, and Y_A) were transcribed, but the proportion was dramaticlly decreased to 40 % in the biofilm niche in the anoxic tank (A_L) (Fig. 2b). NXR complex is an important part for nitrite-oxidizing bacteria associated with oxidizing nitrite to nitrate, which is composed by three subunits including NxrA, the main catalytic units, NxrB and NxrC responsible for converting electrons released by nitrite oxidation to cytochrome c (Lücker et al. 2010). As expected, genes conding for NXR complex were encoded and their transcripts were detected in the two novels *Ca.* Nitrospira MAGs (Table S3). In detail, one candidate *nxr*AB and two *nxr*C were identified in MAG of NOB01, while two copies of *nxr*A, one *nxr*B, and one *nxr*C were recovered from MAG of NOB02. NxrA of NOB01 and NOB02 shared a 98.98 % amino acid identity, while the two copies of NxrA in NOB02 had a 97.91 % amino acid identiy.

To better elaborate the function of NxrA, we select the nearly full-length *nxr*A as a representative sequence for further genetic function analysis. One [Fe-S] cluster and one molybdenum binding motif were identified in NxrA, likely suggesting NxrA of *Ca.* Nitrospira belongs to the Mo-co-binding enzymes in the dimethyl sulfoxide (DMSO) reductase family (Fig. S3a). Consistent with the NxrA in *Nitrospira defluvii* (Lücker et al. 2010), five potential substrate entry regulation channels or NO_2_^−^/NO_3_^−^ catalytic active amino acid sites associated with substrate-binding during nitrite oxidation were identified, in which one threonine active binding site was replaced by asparagine (Fig. S3a). Additionally, four cystenine-rich binding motifs of [Fe-S] clusters related to intramolecular electron transfer were characterized in the NxrB of *Ca.* Nitrospira (Fig. S3b), which has been found in the NxrB of *Nitrobacter* and *Nitrococcus*, and the NarH of *E. coli* (Lücker et al. 2010; Ushiki et al. 2018). NxrC might act as membrane anchors and transfer electrons received from the β-subunit to the electron transport chain via one or two hemes (Rothery et al. 2008; Ushiki et al. 2018). Phylogenetic analysis suggests the *nxr*A of *Ca.* Nitrospira has high affiliation with the periplasmic *nxr*A from *Nitrospira* (Fig. S3a)*.* Further analysis found that NxrA of *Ca.* Nitrospira contains an N-terminal signal peptide to export protein via the twin-arginine protein translocation (Tat) pathway (Kitzinger et al. 2018; Palmer and Berks 2012), and NxrC encode an N-terminal signal peptide for translocation via Sec pathway (Kitzinger et al. 2018), demonstrating that NxrA of *Ca.* Nitrospira was anchored in the periplasmic space and catalyzed an energetically advantageous nitrite oxidation reaction, and the produced protons can be directly released into the periplasm and contribute to proton motive force (PMF) (Kitzinger et al. 2018; Lücker et al. 2010). *Ca.* Nitrospira highly transcribed genes of NXR complex in all four samples for NO_2_^−^ oxidation, and the released NO_3_^−^ could be further utilized by the coexisting denitrifying bacteria (Fig. 2c).

**Fig. 3:**
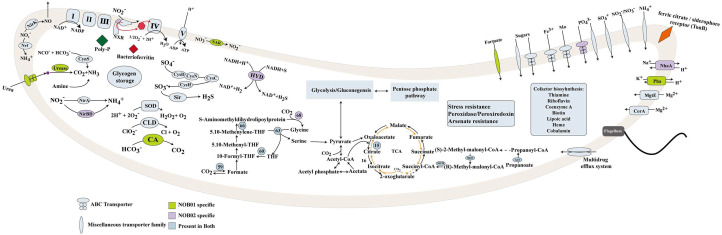
**Metabolic cartoon constructed from the annotation of the two nearly completely sequenced NOB01 and NOB02 genomes and the metatranscriptomic data.** Numbers and gene abbreviations at partial pathway match the enzyme identifiers in Table S3.

**Fig. 4:**
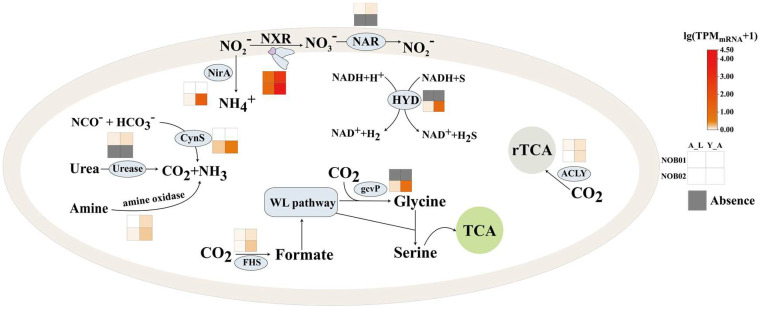
Metabolic activitis of the two novel NOB in different niches.

These two VII *Ca.* Nitrospira strains encoded a copper-containing nitrite reductase (NirK) for nitrite reduction to NO and the transcription of *nirK* was detected (Fig. 2c, Table S3). The presence of NirK occurs in all sequenced *Nitrobacter, Nitrococcus, Nitrospira*, and *Nitrospina* genomes, suggesting that these NOB can produce NO (Daims et al. 2016; Koch et al. 2015). The produced NO can not be further reduced by *Ca.* Nitrospira because of the absence of nitric oxide reductase (NOR). NO is an essential intermediate of ammonia oxidation in AOA, AOB, and anammox bacteria (Caranto and Lancaster 2017; Hu et al. 2019; Kozlowski et al. 2016). Therefore, we proposed that the released NO by NOB01 and NOB02 in the aggregates could modulate the growth and the metabolism of the coexisted anammox bacteria and AOB/AOA and form a mutualistic interaction (Fig. 2c, Table S3). The two subunits of *nrfAH* were detected in NOB02, while only *nrfH* was found in the recovered part of NOB01 with slightly transcribed (Fig. 3), suggesting that nitrite could also be catalytically reduced to ammonium by periplasmic cytochrome c nitrite reductase (Simon et al. 2000; Simon et al. 2001; Ushiki et al. 2018). It has been suggested that nitrite reduction in *Nitrospira* was carried out by a cytoplasmic ferredoxin-nitrite reductase (NirA) for assimilation purpose rather than NrfAH (Daims et al. 2015; Koch et al. 2015; Lücker et al. 2010; Ushiki et al. 2018), and NirA also was identified in NOB01 and NOB02 genome. Thus, the pathway of assimilatory nitrite reduction to ammonium may operate as a result of the transcription of NirA (Fig. 3, Fig. 4).

The retainment of a membrane-bound nitrate reductase complex (NAR, NarGHI) was recently reported in two comammox *Nitrospira* genomes, *Ca.* Nitrospira sp. LK70 and *Ca.* Nitrospira sp. HKST-UBA10, and inferred to be associated with nitrate reduction under anoxic conditions (Wang et al. 2021; Yang et al. 2020a). Interestingly, the NAR complex was encoded by NOB01 and exhibited transcriptional activity. This finding was unexpected, because NOB was proposed to use their NXR for catabolic nitrate reduction (Ehrich et al. 1995; Koch et al. 2015). From a bioenergetic perspective, the cytoplasmically oriented NAR could be a more efficient nitrate reductase than NXR. The presence of NAR likely confer a selective advantage to nitrate-reducing NOB01 over other NOB strains in anoxic conditions .

**Fig. 5:**
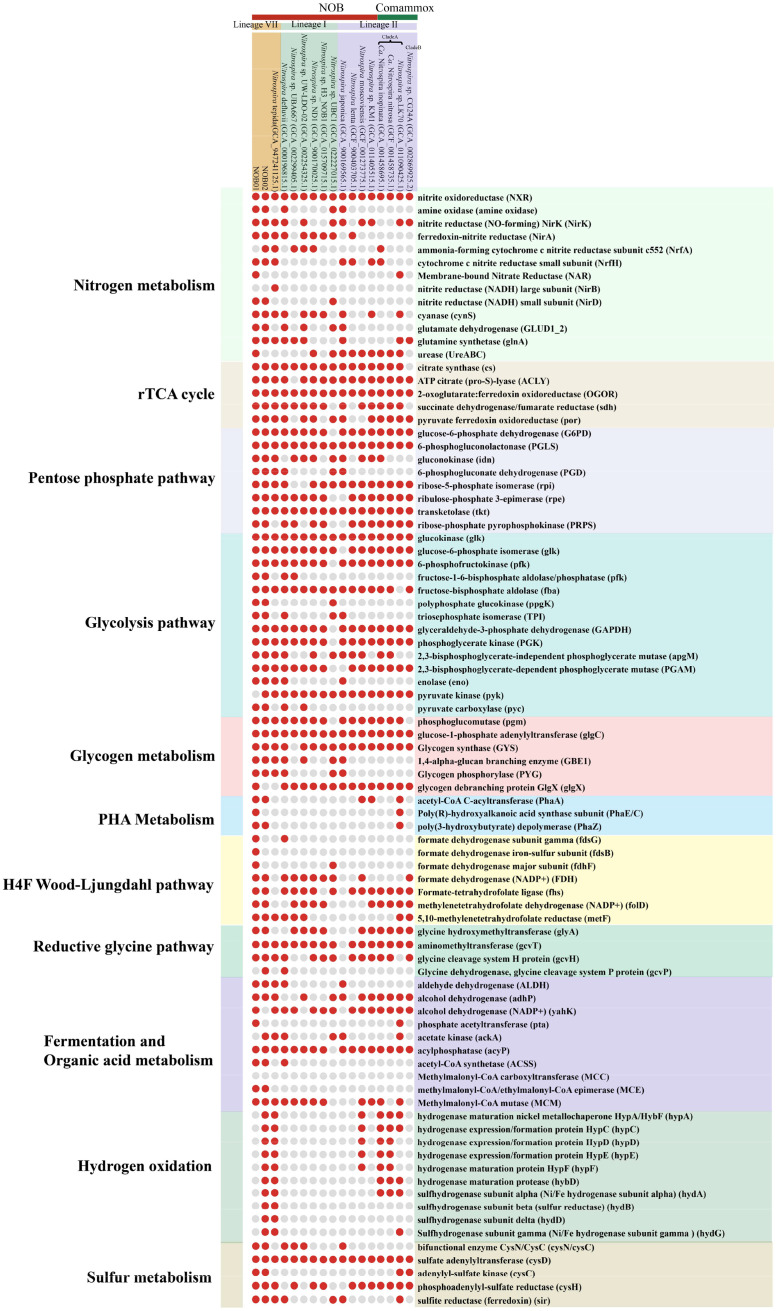
The distribution of key marker genes related to different metabolisms in NOB and Comammox *Nitrospira* genomes.

Another feature that makes *Ca.* Nitrospira attractive is that encoding of urease (UreABC) and cyanase (CynS), the two enzymes that catalyze the remineralization of organic matters to ammonium. Such enzymes also were discovered in *Nitrospira* and were considered to play an significant role. For example, urea transporters and cytoplasmic ureases were discovered in several *Nitrospira* genomes (Fig. 5), and the experimental results further directly confirm the ability of hydrolyses urea in *N.* moscoviensis (Daims et al. 2016; Koch et al. 2015). Also, NOB can better adapt to the scarce free ammonia condition by using urea as an energy source (Daims et al. 2016; Koch et al. 2015). Well-characterized cyanase also was identified in *N.* moscoviensi and catalyzes the reaction of cyanate with bicarbonateto produce ammonium for providing an alternative source of ammonium (Koch et al. 2015). Indeed, alternatively ammonium sources are not only an important survival strategy for NOB, but also allow it to form a mutual aid relationship with nitrifiers in the community (Daims et al. 2016; Koch et al. 2015). Studies have pointed out that urease-positive or/and cyanase-positive NOB can provide urease-negative or cyanase-lacking ammonium-oxidizing bacterium, such as *Nitrosomonas* europaea and *Nitrosomonas nitrosa* Nm90 with ammonia released from urea and cyanate degradation (Koch et al. 2015; Palatinszky et al. 2015). Again, this reciprocal feeding of NOB and AOM interaction also play a key role in co-existence system, because AOM produce nitrite, which is required assubstrate and also detoxified by NOB (Daims et al. 2016). Urease and cyanase were transcribed in retained *Ca.* Nitrospira (Fig. 4) demonstrated that they have the ability of urea and cyanate degradation, and likely forming a reciprocal feeding relationship with ammonia oxidizers, such as *Nitrosomonas europaea*, which was the prodomination AOM in our studied WWTP (Table S4), and the cleavage of urea and cyanase by *Ca.* Nitrospira likely illustrated the initial step that trigger nitrification by *Ca.* Nitrospira-AOM consortia.

### Mixotrophic and alternative metabolism of *Ca*. Nitrospira

Pathways that related to organic substrates catabolic degradation and the assimilation of pyruvate, formate, acetate, and propanoate were encoded by the two *Ca*. Nitrospira genomes (Fig. 3). Since the gluconeogenesis and glycolysis (Embden-Meyerhof-Parnas, EMP) pathway is nearly complete (Table S3, Fig. 3), operation of the sugars catabolism in NOB01 and NOB02 was confirmed. The coding of mixotrophic metabolic pathway can help NOB01 and NOB02 survive in complex full-scale wastewater treatment systems and switch lifestyles with environmental changes (Lücker et al. 2010, Yang et al. 2020). *Ca*. Nitrospira contains the majority of genes related to oxidative tricarboxylic acid (oTCA) only except for the absence of 2-oxoglutarate dehydrogenase complex (ODH) (Table S3, Fig. 3), which was considered an alternative energy generation pathway in *Nitrospira* (Lücker et al. 2010). It was suggested that 2-oxoglutarate: ferredoxin oxidoreductase (ODOR) might replace ODH for 2-oxoglutarate dehydrogenase in *Nitrospira* (Lücker et al. 2010), and genes coding ODOR were identified in MAGs NOB01 and NOB02. However, such alternative strategy is only based on speculation of genomic information, because OGOR can replace the missing ODH in *Helicobacter pylori* (Lücker et al. 2010). Additionally, enzymes related to pentose phosphate pathway and galactose metabolism also were encoded by MAGs NOB01 and NOB02 for sugar catabolism (Table S3).

The existing genome data indicate that NOB *Nitrobacter* and *Nitrococcususe* encode the Calvin–Benson–Bassham (CBB) cycle for CO_2_ fixation (Starkenburg et al. 2006). Differently, NOB *Nitrospira* use CO_2_ as their carbon source through the reductive tricarboxylic acid (rTCA) cycle, and the key enzymes of the rTCA cycle are ATP citrate (pro-S)-lyase, 2-oxoglutarate: ferredoxin oxidoreductase (ODOR) and ferredoxin oxidoreductase (POR) (Lücker et al. 2010). Encoding of these genes suggesting NOB01 and NOB02 also fix CO_2_ via a typical rTCA cycle (Fig. 3). Interestingly, POR and OGOR are O_2_ sensitive enzymes, and in *Nitrospira*, a unique five subunit leads to better oxygen tolerance of these two enzymes (Lücker et al. 2010; Mueller et al. 2023), which helps *Nitrospira* adapt to the rTCA metabolic pathway in trace oxygen for survival. Recently, a metabolic pathway named the reductive glycine pathway (RGP) was constructed and found to be involved in CO_2_ fixation in *Candidatus* Phosphitivorax (Figueroa et al. 2018; Tashiro et al. 2018). Subsequently, an interconnected process Wood–Ljungdahl (WL) pathway and RPG has also been confirmed to fix CO_2_ in *Clostridium drakei* by ^13^C isotope-based metabolite-tracing experiments (Song et al. 2020). Indeed, the bottom line genes that are related to WL and RPG pathway, such as amino methyltransferase (*gcv*T), glycine cleavage system H protein (*gcv*H), hydroxymethyltransferase (*gly*A/ SHMT), glycine dehydrogenase (*gcv*P), formate dehydrogenase (NADP+) subunit (*fdh*), formate–tetrahydrofolate ligase (*fhs*), methylenetetrahydrofolate dehydrogenase (NADP^+^) (*fol*D) and methylenetetrahydrofolate reductase (*met*F) were identified in NOB01, while NOB02 lack the key gene glycine dehydrogenase (*gcv*P) involved in the most critical step of the RPG pathway (Fig. 3, Fig. 5). Thus, NOB01 has the genetic potential to fix CO_2_ through the RPG and WL pathway (Table S3, Fig. 3). Previous research has found that the encoding of the RGP pathway enables *Desulfovibrio deulfuricans* to achieve autotrophic growth, and in the presence of sufficient ammonia, the growth rate of autotrophic *Desulfovibrio deulfuricans* increases with the increase of ammonia (Sánchez-Andrea et al. 2020). Therefore, it was speculated that the additional CO_2_ fixation pathway likely helps NOB01 survive unfavorable conditions and facilitate switches between lifestyles in fluctuating environments. The presence of RPG pathway regulation genes also were reported in some *Nitrospira* genomes (Fig. 5) (Lücker et al. 2010; Lawson et al. 2021; Ushiki et al. 2018; Yang et al. 2020a). Potential genes of polyhydroxyalkanoates (PHA) biosynthesis and degradation were identified in NOB01 and NOB02, including acetyl-CoA C-acetyltransferase (*pha*A), poly(*R*)-hydroxyalkanoic acid synthase subunit E (*pha*E) and poly(3-hydroxybutyrate) depolymerase like protein (*pha*Z) (Fig. 3, Fig. 5). Poly(*R*)-hydroxyalkanoic acid synthase subunit C (*pha*C) and acetoacetyl-CoA reductase (*pha*B) were absent probably due to the incomplete nature of genome of NOB01 and NOB02 (McCool and Cannon 2001; Parveez et al. 2015; Zher Neoh et al. 2022).

H_2_ and formate are two common products at oxic/anoxic interfaces, and putatively involved in alternative energy metabolisms under low O_2_ environment (Bayer et al. 2021). Recently researches suggested that some *Nitrospira* members possess the ability to utilize other than nitrite and CO_2_ as energy source. The first chemolithoautotrophic lifestyle independent of the nitrogen cycle was *N. moscoviensis* which encodes a group 2a [NiFe] hydrogenase and accessory proteins, and experimental result further comfirmed *N.* moscoviensis that can be achieved energy conservation via aerobic oxidizing H_2_, and can grow with H_2_ as the sole energy source and electron donor (Koch et al. 2015, Leung et al. 2022). [NiFe] hydrogenase also were identified in the genomes of *Nitrospira* (Bayer et al. 2021; Yang et al. 2020a) and *Nitrospina* gracilis (Luecker et al. 2013). Furthermore, *N.* moscoviensis also can couple formate oxidation to NO_3_^−^ reduction to obtain energy under various O_2_ concentration condition, and formate dehydrogenase is the main function enzyme (Koch et al. 2015). A group 3b hydrogenases (HYD) and formate dehydrogenase were uniquely encoded with slightly transcription by *Ca.* Nitrospira (Fig. 5, Fig. S6), likely enabling *Ca.* Nitrospira to use H_2_ and formate as an alternative energy source in the biofilm and activated sludge niche.

### Interaction of VII *Ca*. Nitrospira with other microbes

Suppressing NOB growth is a key element for removing nitrogen in anammox-based process, and many factors such as high temperature, high ammonium/ammonia concentration (Ganigué et al. 2007), high nitrite/ free nitrous acid concentration (Wang et al. 2014), and especially low dissolved oxygen (DO) concentration (Ma et al. 2015), have been identifed effectively inhibit or limit NOB growth. The relative abundance of *Ca*. Nitrospira and NXR activity, however, had no remarkable inhibition in the two anaerobic tanks (1_A and 2_A) with low DO concentrate (Fig. S2), Indeed, the recycled wastewater from the oxic tank (2_O, DO: 5.01 mg/L) might bring trace amounts of oxygen into the two anaerobic tanks (1_A and 2_A), which likely provided a promising opportunity for growth of *Ca*. Nitrospira. Interestingly, the abundance of *Ca.* Nitrospira was dramatically decreased in the anoxic tank (Y_A and A_L), in which anammox bacteria were highly enriched (Fig. 2a). The restricted oxygen diffusion leads to a relatively low DO microenvironment in biofilm (Cho et al. 2020; Ma et al. 2015). The low DO resulted in the low activity of NOB due to the unavailability of sufficient oxygen and securied a strong competitive edge for anammox bacteria. Furthermore, an obvious competitive relationship occurs in *Ca*. Nitrospira, anammox bacteria and denitrifers also seems to provide clues to the suppression of *Ca*. Nitrospira (Fig. 3c& d). Nitrite, as the growth substrate for *Ca*. Nitrospira, was heavily occupied by anammox bacteria and denitrifiers, because high transcriptional activities of *nxr* and *nirK* in anammox bacteria leading the mainstream NO_2_^−^ was rapidly convert to NO_3_^−^ and NO. Also, part of NO_2_^−^ could be reduced to NO by denitrifiers due to the transcription of their *nirK* (Fig. 2).

Indeed, although the growth of *Ca.* Nitrospira was inhibited, it non-negligibly maintains the community's nitrogen transfer and construct a reciprocal feeding network. As mentioned above, encoding some ammonium generation enzymes, UreC CynS and amine layse, confer *Ca.* Nitrospira the ability to release ammonium into the community for feeding the AOM (Daims et al. 2016). Also, *Ca.* Nitrospira and denitrifying bacteria in the sludge (Y_A) also highly transcribed *nirK* (Fig. 3c), but most of them lack the nitric-oxide reductase (NOR) enzyme for further NO reduction, which likely resulted in a high amount of NO release from sludge. NO is a key intermediate in microbial interaction (Daims et al. 2016; Hu et al. 2019), thus, the released NO in the community might be directly used by anammox bacteria as the electron acceptor for anaerobic ammonium oxidation (Kartal et al. 2010) or could diffuse into the biofilm system to support the enrichment of anammox bacteria. In addition, NO released by *Nitrospira*-NOB may recruit AOB (such as in *N*. europaea) to form nitrifying aggregates, which helps to promote the formation of anaerobic cores and the enrichment of anammox bacteria. The detectable expression of NarG by NOB01 indicates that some NOB01 could respiration of organic substrates with nitrate as the terminal electron acceptor, and the released NO_2_^−^ could be mainly utilized by denitrifiers and anammox bacteria (Fig. 3c).

### NAR and NXR in *Ca*. Nitrospira

**Fig. 6:**
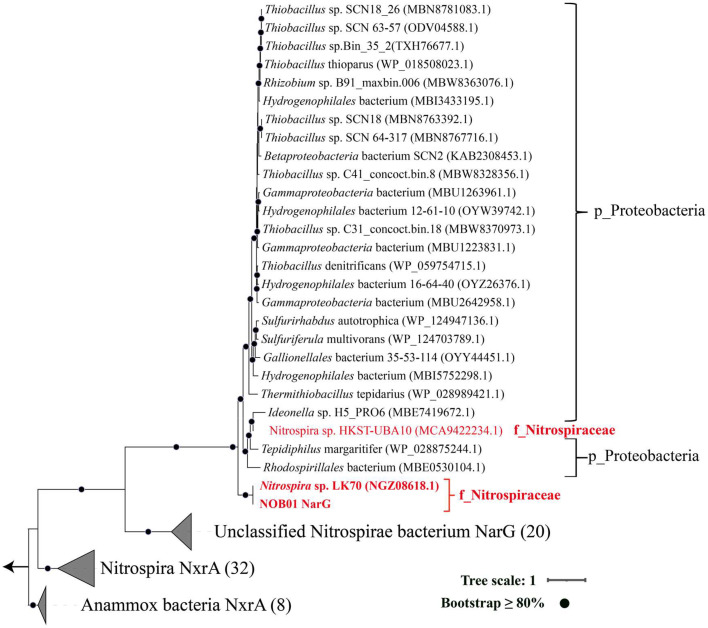
**Maximum likelihood tree highlights the affiliation of nitrate reductase (NarG) recovered from NOB01 in this study and other NarG and NxrA**, the tree was rooted by NxrA from anammox bacteria.

Membrane-anchored nitrate reductase (NAR), which belongs to the family of molybdenum (Mo)-containing DMSO reductases (Coelho and Romão 2015; González et al. 2006) and is often absent in *Nitrospira*-NOB, was annotated in the genome of NOB01. NAR complex has been discovered in many aerobic microorganisms, for example, in the model organism *Escherichia coli*. Unlike with NXR, the NAR complex is a trimeric enzyme, NarG and NarH are situated in the cytoplasm and NarI anchors to the membrane. Analogous cases are also discovered in some bacteria, such as *Marinobacter hydrocarbonoclasticus, Paracoccus pantotrophus* (Coelho and Romão 2015; Marangon et al. 2012). In all characterized *Nitrospira* genomes, only two *Nitrospira* (*Nitrospira* sp. LK70, and *Nitrospira* sp. HKST-UBA10) encode the NAR complex (Fig. 6) (Wang et al. 2021; Yang et al. 2020b), but the function was often elusive. Therefore, we first try to elucidate the potential function of the NAR complex in NOB01 based on a homologous modeling approach. Owing to the conserved structure of the NAP complex, thereby the three-dimensional structure of the NAR complex of NOB01 was modeled homologously based on existing models of the NAR complex (Fig. 6, default search for the best model). The best-matched model is 3EGW (PDB ID) (Gonzalez et al. 2017) and the quality related parameters such as GMQE (0.9) and QMEANDisCo Global (0.83 ± 0.05)were the expected result, suggesting model was constructed with good quality and high confidence (Studer et al. 2019; Waterhouse et al. 2018). The NAP complex of NOB01 also belongs to the DMSO reductases, in which the catalytic subunit NarG is the molybdopterin-dithiolenes (Mo-*bis*MGD) cofactor and an iron-sulfur center of the [4Fe-4S] type (FeS0) enzyme (Fig. S5b) which shares the same Mo-*bis*MGD and FeS0 binding sites with the NarG of *E. coil* (Fig. S5b) (Bertero et al. 2003). Five NO_2_^−^/NO_3_^−^ catalytic active sites were encoded for biocatalytic nitrate reduction (Fig. S5). In *E. coli*, the NarH is the electron transfer subunit and contains three [4Fe-4S] (SF4) and a [3Fe-4S] (F3S) center, such as PDB ID: 1Q16, or two [4Fe-4S] (SF4) and two [3Fe-4S] (F3S) center like PDB ID: 3EGW, the small NarI subunit contains two b-type hemes cofactors and hooked NarGH at the cytoplasmatic side of the membrane, providing the binding site for the oxidation of electron quinol. As previously proposed operation process of nitrate reduction, menaquinone (MQH_2_) located on the cytoplasmic membrane is initially oxidized by NarI with the release of two electrons, which are subsequently transferred to NarG via NarH to activate the reduction of nitrate (Coelho and Romão 2015; González et al. 2006), suggesting the entire nitrate reduction process requires the collaboration of the three subunits. Comparably, the potential binding site of SF4, F3S, and heme were found in the NarHI sequence of NOB01 (Fig. S5c & d), but some amino acid residues are different with the NarI of *E. coil*. Intriguingly, although the binding sites of the two heme (the low-potential heme bL and the high-potential heme bH) of NarI in NOB01 differ from those of E. coil, the binding site of the histidine groups (heme bH coordinated with His^56^ and His^205^, and ferrous heme bL coordinated with His^66^ and His^187^) of the Fe atoms involved in the transfer of electrons is conserved (Fig. S5e). Thus, we concluded that the NAR complex of NOB01 should be capable of catalyzing the reduction of NO_3_^−^ to NO_2_^−^. But the extent to which this affects the electron transfer efficiency and the biochemical processes mediated by NarHI subunits need further *in-vitro* verification.

Although both NXR and NAR belong to (Mo)-containing DMSO family enzymes, they seemly undergo different evolutionary histories. Previously studies suggested that the *nxr*A of *Ca.* N. defulvii has the closest affiliation to *nxr*A of *Ca.* Kuenenia stuttgartiensis (Daims et al. 2015; Lücker et al. 2010), and inferred the potential horizontal gene transfer (HGT) between anammox bacteria and *Nitrospira*. Herein, we also conducted the phylogenetic analysis of NxrA sequences of *Ca.* Nitrospira (Nitrospiraceae), *Nitrospira*, anammox bacteria, *Nitrotoga,* and *Nitrobacter* (Fig. S3b). The most adjacent branches of the *nxr*A cluster of *Ca.* Nitrospira and *Nitrospira* were anammox bacteria, which coincides with previous phylogenetic analysis (Daims et al. 2015; Kitzinger et al. 2018) and likely illustrates the *nxr*A of *Ca.* Nitrospira also evolved from anammox bacteria. In accordance with the result of *nxr*A, the closest homolog of *Ca.* Nitrospira *nxr*B was in anammox bacteria (Fig. S3d), and the closest homologous *nxr*B of Nitrospiraceae is *Scalindua*, which further proof of the speculation that NXR of the Nitrospiraceae was originated from anammox bacteria. Here, we speculated that the NXR of *Ca.* Nitrospira and *Nitrospira* might inherite from a common ancestor which acquired the NXR from anammox bacteria through HGT in the early evolution stage of these two lineages.

Intriguingly, comparing genomics revealed an unexpected evolutionary link between Nitrospirae and Proteobacteria phylum in the NAR complex. Phylogenetic analysis revealed that NarG of *Ca.* Nitrospira sp. NOB01 and *Nitrospira* sp. LK70 had higher affiliation with bacteria in Proteobacteria compared to other *Nitrospirae* (Fig. 6), which may indicate the quite different evolutionary sources of NarG in phylum *Nitrospirae*. NarG seuqences from NOB01 and *Proteobacteria* shared an over 60% amino acid sequence identity, which is much higher than with *Nitrospirae* (less than 49%), reflecting the HGT of NarG was occured between *Ca.* Nitrospira and *Proteobacteria*. Consistently, the high identities of NarH, NarI, and NarJ between NOB01 and *Proteobacteria* were also found (Table S4).

### Stress defense

Many genes associated with coping with environmental adversities were detected in NOB01 and NOB02. Superoxide dismutase (SOD), thioredoxin-dependent peroxiredoxin (prx/tpx) (ref), and thioredoxin reductase (txrAB) (ref) related to oxygen resistance were found in NOB01 and NOB02 (Table S4). NOB01 has a more reactive oxygen species (ROS) scavenging system, which composed by superoxide dismutase (SOD), thioredoxin-dependent peroxiredoxin (prx/tpx) and thioredoxin reductase (txrAB), than NOB02, confers a survival advantage to NOB01 in the fluctuating wastewater systems (Y_A and Y_B). Resistance-nodulation-division (RND) system related genes *acr*AB and *mtd*ABC were recovered in NOB01, while *mtd*ABC was lacking in NOB02, likely suggesting NOB01 can be very effective in counteracting various toxic antibiotics, detergents, and biocide (Du et al. 2014; Hadchity et al. 2021; Nuonming et al. 2018). Additionally, heavy metals resistance genes, including *cus*ABC efflux system and arsenite reductase (*ars*C) were encoded by NOB01 and NOB02, which potentially enhance their tolerance to heavy metals in sewage. Chlorite dismutase (CLD) often occurs in other NOB, including *Nitrospira* and *Nitrobacter* for chlorite detoxification (Daims et al. 2015; Lücker et al. 2010; Mlynek et al. 2011; Yang et al. 2020a), which also was found in NOB01 and NOB02. Interestingly, two identical locus coding for CLD were found in NOB02, whihc likely helps NOB02 to survive in the anoxic biofilm systems, since the producted oxygen by CLD could be used for aerobic respiration (Hofbauer et al. 2014; Kostan et al. 2010; Yang et al. 2020b). High-salinity concentrate is a common problem for many sewage, which may decrease the effectiveness of biological wastewater treatment *via* charging the microbial activity (Zhao et al. 2020). Na^+^/*H*^+^ antiporter (NhaA and NhaK), magnesium transporter (CorA), magnesium transporter (MgtE), and multicomponent *K*+:*H*+ antiporter complex (Pha) (Assaha et al. 2017; Sun et al. 2022) related to salt stress tolerance were found in the two MAGs. Clustered Regularly Interspaced Short Palindromic Repeats (CRISPRs), together with CRISPR-associated (Cas) proteins, confer adaptive immunity to microbials in their defense against unwanted environment (Marraffini and Sontheimer 2010). Multiple families of *Cas* proteins also build unique CRISPR-Cas immune systems for microbes, for example, Csn-type systems have been shown to cleave invasive heterologous DNA (Garneau et al. 2010), and DNA silencing was mediated by Csm-type Cas proteins (Marraffini and Sontheimer 2010), and the Cmr-type complex are involved in the recognition and destruction of complementary RNAs in vivo (Hale et al. 2012). Some CRISPR-Cas immune systems regulation genes also were found in NOB01 and NOB02, especially in NOB01, which encode various Cas genes, this result suggests that NOB01 and NOB02 can effectively resist phage infestation.

## Conclusions

After genomic recovery and characterization, we identified two novel VII *Nitrospira* species NOB01 and NOB02, and construct a substantial potential metabolic versatility. The identification of NXR consolidates the metabolic potential of *Ca*. Nitrospira for nitrite oxidation. In particular, the discovery of a urease and cyanase gene indicated *Ca*. Nitrospira can utilize urea and cyanate as substrates for ammonia production, and benefit from this, *Ca*. Nitrospira formed a reciprocal feeding relationship with the AOM in the community for performing nitrification. Our results also indicate that *Ca*. Nitrospira may interact with other members of the microbial communities, such as anammox bacteria and denitrifiers, through the supply of NO and NO_3_^−^, resulting from transcribed *nir*K and *Nar*G in *Ca*. Nitrospira. In addition to classical r-TCA carbon fixation pathway, we also deduced that an interconnected process WL pathway and RPG for extra carbon fixation, and benefit from above carbon fixation pathway and H_2_ and formate metabolism would further increase the ecophysiological flexibility of *Ca*. Nitrospira in activated sludge and biofilm niche. In particular, nitrate reduction coupled with organic carbon oxidation might confer a selective advantage to NOB02 in the activated sludge niche. Our results indicate that coinciding with previous results, the closest relative of *Ca.* Nitrospira NXR is anammox bacteria, likely illustrating the occurrence of HGT. Importantly, the NAR complex only detected two *Nitrospira* genomes and NOB01, and its potential function profiling and evolution history also were illustrated by homology modeling and comparative genomic analysis. In summary, by introducing the novel genus of NOB *Ca*. Nitrospira and charactering their metabolic versality in WWTP system, our findings suggest that the diversity and abundance of NOB may be higher than previously thought. Moreover, there are also other prominent open questions pertaining to this genus of bacteria. Further studies should focus on the enrichment and physiological characterization of the novel NOB, such as the underlying reason for NAR complex coding and the real catalytic function of the NAR complex in *Ca*. Nitrospira.

## Materials and methods

### Sampling collection, sequencing, and binning

Procedures of sample collection, processing, and data generation and analysis are provided in our previous study (Hu et al. 2023). In brief, the activated sludge and biofilm were sampled in a full-scale wastewater treatment plant (WWTP) treating duck breeding wastewater, which was configured as an anaerobic-anoxic-anaerobic-oxic system. Samples were preserved in LifeGuard Soil Preservation (QIAGEN, Germany), and total DNA and RNA in all samples were extracted using the DNA and RNA extraction kits (QIAGEN, Germany) within 24 h. Shotgun metagenomics and metatranscriptomics sequencing were carried out by Novogene Co., Ltd. (Beijing). Raw shotgun metagenomic sequencing reads were quality controlled by MetaWRAP read_qc with default setting (Uritskiy et al. 2018), and retained clean reads were *de novo* assembled by MEGAHIT V1.1.4 (–k-min 23 –k-max 141 –k-step 20) (Li et al. 2015). Scaffolds larger than 1500 bp were binned by metabat2, maxbin2, concoct, and refined by MetaWRAP bin_refinement with the setting “-x 50 -c 50″ (Uritskiy et al. 2018). The quality of obtained MAGs was assessed using the module “lineage_wf” of CheckM v1.0.6 (Parks et al. 2015), and eligible MAGs (completeness > 50 % and contamination <10 %) were manually refined for downstream analysis. All refined MAGs were classified using GTDB-Tk v1.3.0 (Chaumeil et al. 2019) with the version Genome Database Taxonomy GTDB-r214 (Parks et al. 2020) as the database and default setting. Clean metagenomic reads were mapped to the scaffolds by BWA v0.7.17 (Li and Durbin 2009) with the default setting to calculate the genome and recorded gene abundance as TPM. For trimmed metatranscriptomic data, the non-rRNA dataset was constructed by removing the ribosomal RNA (rRNA) reads from raw data using SortMeRNA (version 2.1) (Kopylova et al. 2012) based on the SILVA 132 database. The transcription abundance (TPM) was calculated by mapping all non-rRNA datasets to all predicted ORFs by BWA v0.7.17 (Li and Durbin 2009) with a default setting.

### Genome reassemble and annotation

In total three NOB MAGs were acquired and identified, including NOB01, NOB02, and NOB03. To improve the quality and exclude unexpected contaminations in binning, the two novel NOB MAGs NOB01 and NOB02 were subjected to highly iterated and rigorous reassembly (Yang et al. 2020a, Yang et al. 2021). Briefly, clean reads of the samples oxic tank (2_O) and biofilm (A_L) were mapped to the two NOB MAGs using BBmap (Bushnell 2014), and the successfully aligned reads were re-assembled using SPAdes v3.12.0 (Bankevich et al. 2012) with the k-mers of 21, 33, 55, 77, 99, 127, then, the quality of the resulting MAGs of two NOB was checked using CheckM v1.0.6 “lingage_wf” command again (Parks et al. 2015). The protein-coding genes in the reassembled MAGs NOB01 and NOB02 were predicated using Prodigal v2.6.3 using the “- p single” option (Hyatt et al. 2010). Genome annotation was carried out by GhostKOALA (Kanehisa et al. 2016) using the KEGG database, eggNOG-mapper (Huerta-Cepas et al. 2016). The interest function genes were also further confirmed using BLASTp against the NCBI non-redundant (nr) database. To compare the genome-wide gene expression levels of the two NOB MAGs in the targeted reactor and biofilm, gene transcription abundance showed by TPM was standardized by the genome abundance. Average Nucleotide Identity (ANI) was calculated between the newly recovered NOB MAGs and known *Nitrospira* genome downloaded from NCBI database by fastANI (Jain et al. 2018) with the default setting.

### Phylogenetic analysis

The 43 concatenated marker genes were identified from the newly acquired NOB MAGs and known *Nitrospira* genomes download from NCBI and aligned using CheckM v1.0.6 “lineage_wf” module with default setting (Parks et al. 2015). In addition, a genome-wide *de novo* phylogenetic analysis based on the Genome Database Taxonomy GTDB-r214 database was also performed by GTDB-Tk v1.3.0 “de_novo_wf” command with “-outgroup_taxon P_Nitrospirae” (Chaumeil et al. 2019). The 16S rRNA gene sequences in MAGs NOB01, NOB02, and NOB03 were identified by BLASTn against the SILVA SSU 138 database and their close relatives in genus *Nitrospira* based on BLASTn results and 16S rRNA gene sequences from another characterized NOB genus *Leptospirllum* were downloaded from NCBI. All above 16S rRNA gene sequences were used for the phylogenetic tree built to highlight the phylogenetic placement of the *Ca.* Nitrospira genus in the family of Nitrospiraceae.

For the phylogenies of NxrA and NxrB (nitrite oxidoreductase alpha /beta subunit), the sequence of NOB01 and NOB02 were used as a query in BLASTp search (Mahram and Herbordt 2015) in the NCBI database with the requirements of E-value < 10e-10, amino acid identities > 60 %, and a minimum alignment length > 50 % to find its close relatives. All retained sequences were aligned using MAFFT v7.463 (Katoh et al. 2002) and gaps in alignment were removed using trimAl V2.0 with the setting ‘-gt 0.1′ (Capella-Gutiérrez et al. 2009). Maximum likelihood trees of NxrA and NxrB were constructed using IQ-TREE2 V1.6.12 (Minh et al. 2020) with the LG+R3 and LG+G4 substitution model and 1000 ultrafast bootstraps.

A maximum-likelihood phylogenetic tree of Ni/Fe hydrogenase, using online tools https://services.birc.au.dk/hyddb/ to determine the classification of the sequence, and then, additional Ni/Fe hydrogenase sequences of the family Nitrospiraceae and the genus *Nitrospira* deposited in the NCBI nr-database were also identified using BLASTp (Mahram and Herbordt 2015) using the Ni/Fe hydrogenase sequences of *Ca*. Nitrospira NOB02 as the query and the standard is E-value 〈 10e-10, amino acid identities 〉 60 %, and a minimum alignment length > 50 %. Sequences were aligned using MAFFT v7.463 (Katoh et al. 2002) and gaps in alignment were removed using trimAl V2.0 with the setting ‘-gt 0.1′ (Capella-Gutiérrez et al. 2009). Maximum likelihood trees were constructed using IQ-TREE2 V1.6.12 (Minh et al. 2020) with the Q.pfam+R8 substitution model and 1000 ultrafast bootstraps.

For the phylogeny of NarG (nitrate reductase alpha subunit), the sequence of *Ca*. Nitrospira NOB01 was used as the query in the BLASTp (Mahram and Herbordt 2015) search in the NCBI database (E-value 〈 10e-10, amino acid identities 〉 60 %, and a minimum alignment length > 50 %). To more accurately pinpoint the affiliation of NarG, additional NarG sequences of unclassified *Nitrospirae* bacterium, NxrA of Nitrosipra, and anammox bacteria deposited in the NCBI database were also identified using BLASTp using the NarG sequences of *Ca*. Nitrospira NOB01 as the query and the Evalue of 10e-6. All retained sequences were aligned using MAFFT v7.463 (Katoh et al. 2002) and gaps in alignment were removed using trimAl V2.0 with the setting ‘-gt 0.1’ (Capella-Gutiérrez et al. 2009). Maximum likelihood trees were constructed using IQ-TREE2 V1.6.12 (Minh et al. 2020) with the LG+R4 substitution model and 1000 ultrafast bootstraps. Finally, All the above-built trees were visualized and beautified using iTOL (Letunic and Bork 2021).

### Homologous modeling of NAR complex

NAR complex structure homology modeling was performed by SWISS-MODEL workspace as described in (Bordoli et al. 2009). Briefly, the target NAR amino acid sequence, which is heteromeric and consists of three different protein chains (NarGHI) as subunits, was obtained from “*Ca.* Nitrospira NOB01”, then, the targeted sequence serve as a query to search for evolutionary-related protein structures against the SWISS-MODEL template library SMTL (https://swissmodel.expasy.org/templates/) (Biasini et al. 2014) using BLAST and HHblits (Bordoli et al. 2009; Remmert et al. 2012; Waterhouse et al. 2018). The three-dimensional NAR complex protein model was generated by aligning the first transfer ring conserving atoms to the template, and residue coordinates corresponding to insertions/deletions in the alignment also generated by loop modeling, the final three-dimensional NAR model was constructed by integrating the non-conserved amino acid side chains (Waterhouse et al. 2018). To quantify the modeling error and estimate the expected model accuracy, the expected quality of the resulting models was estimated by Global Model Quality Estimate (GMQE) and Quaternary Structure Quality Estimate (QSQE) (Bordoli et al. 2009; Waterhouse et al. 2018). Additionally, the NAR model also was further evaluated by SAVES v6.0 (Colovos and Yeates 1993; Pontius et al. 1996). Visualization and retouching of the model were carried out by PyMOL v 2.3.2 (Yuan et al. 2016). Cluster analysis of targeted NAR with the template was produced from SWISS-MODEL and PyMOL align module with default setting. Display for the alignment was generated using the ESPRIPT 3.0 tool (http://espript.ibcp.fr/).

## CRediT authorship contribution statement

**Pengfei Hu:** Writing – original draft, Validation, Formal analysis, Data curation. **Youfen Qian:** Writing – original draft, Validation, Investigation. **Yanbin Xu:** Investigation, Data curation. **Adi Radian:** Writing – review & editing, Formal analysis. **Yuchun Yang:** Writing – review & editing, Validation, Formal analysis. **Ji-Dong Gu:** Writing – review & editing, Writing – original draft, Supervision, Conceptualization.

## Declaration of competing interest

The authors declare that they have no known competing financial interests or personal relationships that could have appeared to influence the work reported in this paper.

## Data Availability

Raw metagenomic and metatranscriptomic data have been deposited into NCBI under the BioProject PRJNA815463. All NOB genome sequences from the current study have been deposited in the NCBI GenBank database, with accession numbers JANRMO000000000, JANRMP000000000, and JANRMQ000000000.

## Appendix Supplementary materials

Image, application 1

Image, application 2
