# Supplementary material for: A positive contribution to nitrogen removal by a novel NOB in a full-scale duck wastewater treatment system
Source: Water Res X. 2024 Jul 10;24:100237. doi: 10.1016/j.wroa.2024.100237 (PMC11327836; doi:10.1016/j.wroa.2024.100237)
Supplement: Supplementary file 2 [file mmc2.docx]

**Supplementary materials**

*Water research X*

**A positive contribution of a novel NOB to nitrogen removal in a full-scale duck wastewater treatment system**

Pengfei Hu^a,b^, Youfen Qian^a,b^, Yanbin Xu^c^, Adi Radian^a^, Yuchun Yang^d^, Ji-Dong Gu^b,e,*^

^a^ Civil and Environmental Engineering, Technion – Israel Institute of Technology, Haifa 320003, Israel

^b^ Environmental Science and Engineering Research Group, Guangdong Technion -

Israel Institute of Technology, 241 Daxue Road, Shantou, Guangdong 515063, The

People’s Republic of China

^c^ School of Environmental Sciences and Engineering, Guangdong University of Technology, Guangzhou, Guangdong 510006, The People’s Republic of China

^d^ State Key Laboratory of Biocontrol, School of Ecology, Sun Yat-sen University, Guangzhou, Guangdong 510275, The People’s Republic of China

^e^ Guangdong Provincial Key Laboratory of Materials and Technologies for Energy Conversion, Guangdong Technion - Israel Institute of Technology, 241 Daxue Road, Shantou, Guangdong 515063, The People’s Republic of China

*Corresponding author

Ji-Dong Gu, Tel.: (+852) 2299-0605; fax: (+852) 2559-9114; e-mail: jidong.gu@gtiit.edu.cn


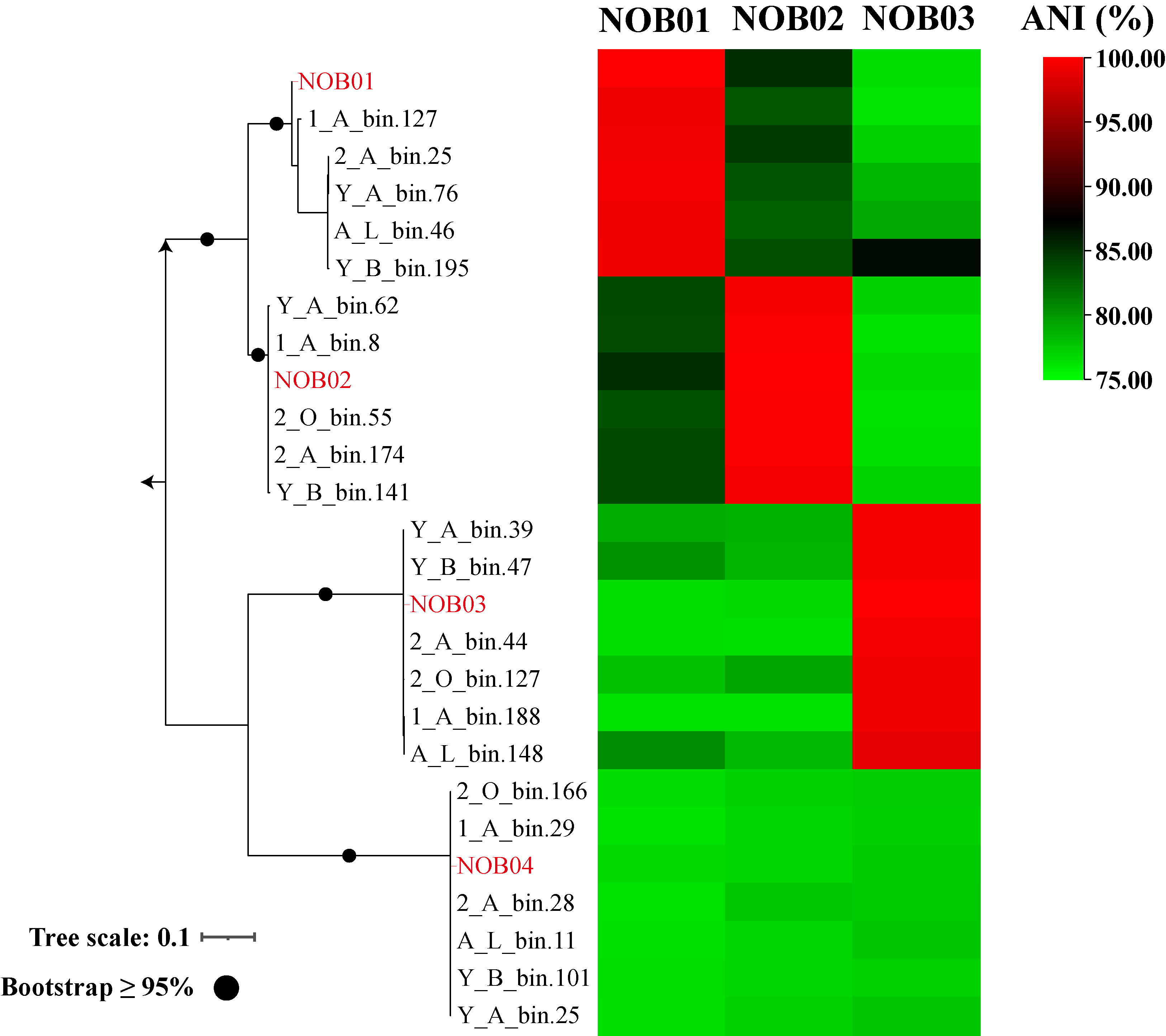


**Figure S1 Phylogenetic analysis and average nucleotide identity (ANI) of the MAGs obtained for NOB.** Tree was rooted by midpoint.


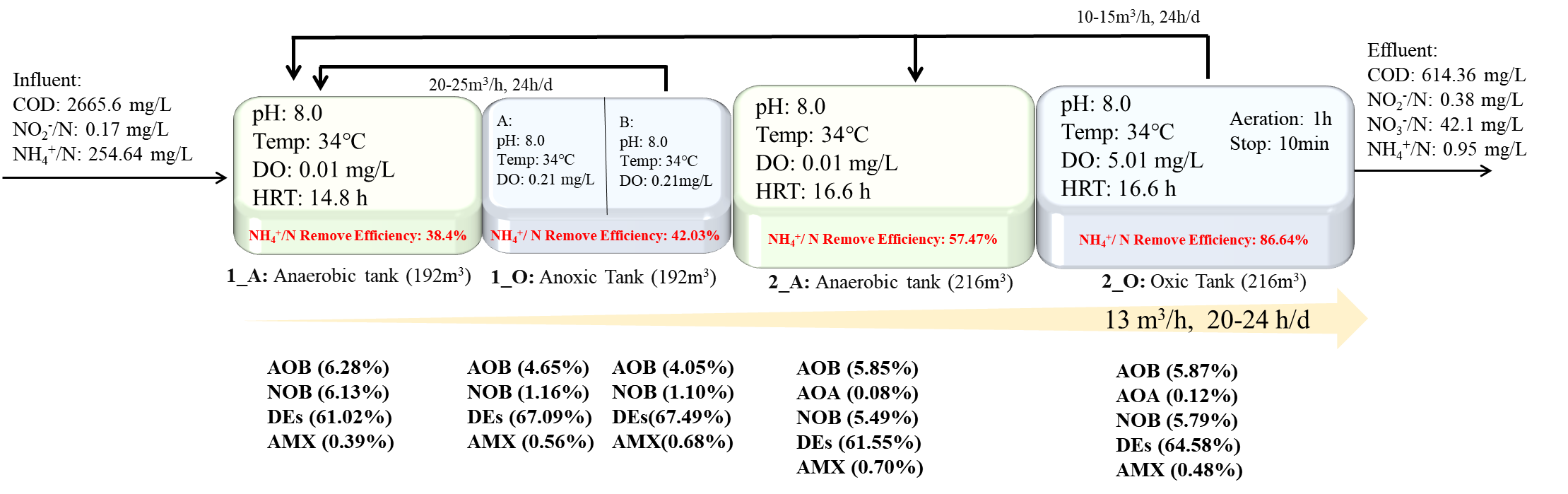


**Figure S2 Workflow of the WWTP.**

Temp: Temperature, DO: Dissolved Oxygen, HRT: Hydraulic Retention Time. COD: Chemical Oxygen Demand. 24 h/d represent the system operates all day long.


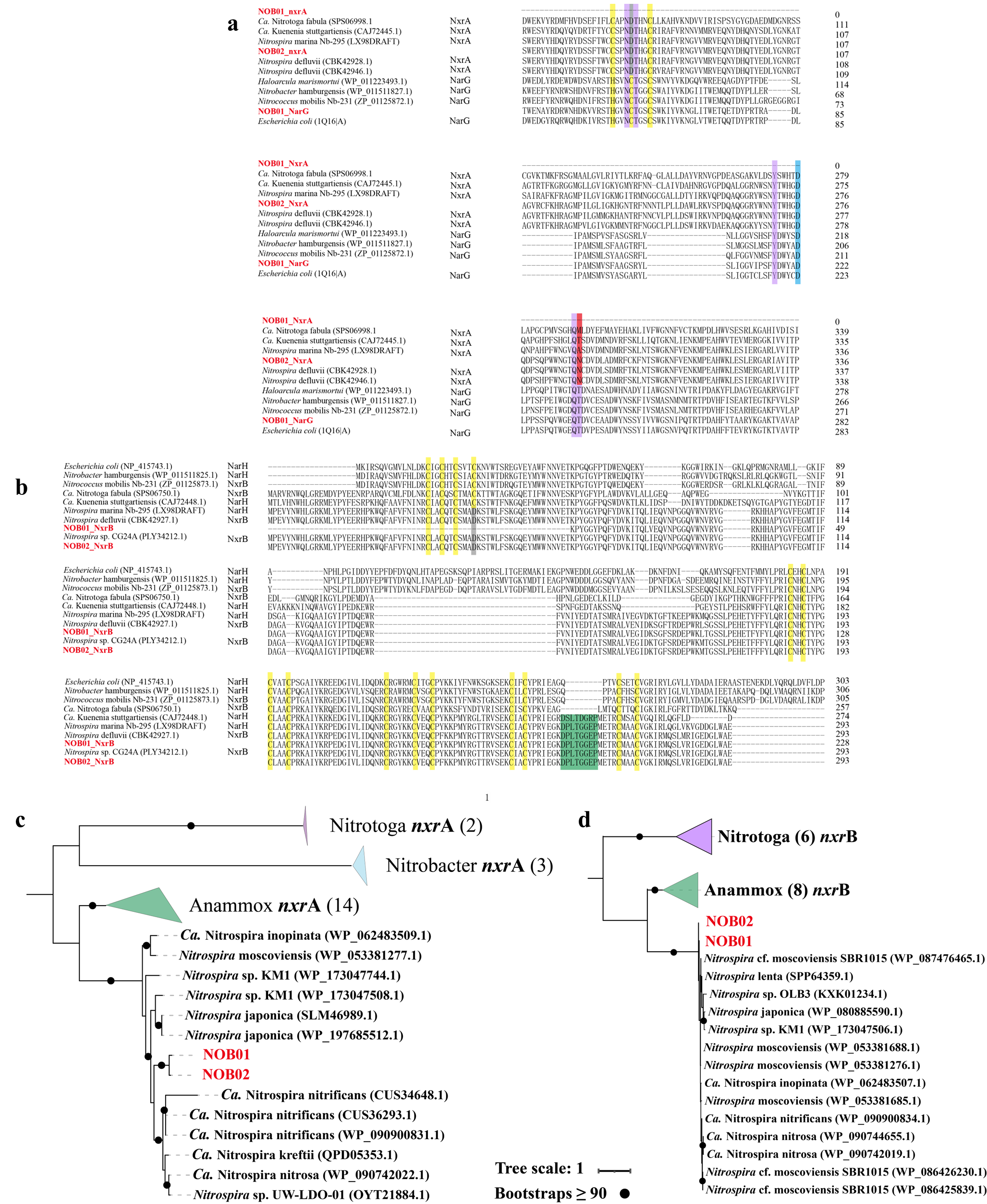


**Figure S3 (a and b): Alignments of metal-coordinating regions in the α-subunits of selected molybdopterin-binding enzymes in the dimethyl sulfoxide (DMSO) reductase family.** (a): Five signature residues, which are conserved in nitrate reductases and nitrite oxidoreductases, are highlighted in purple, and Asparagine (highlighted in red) replaces one threonine signature residue in NxrA of NOB02 and *Ca.* N. defluvii. Iron-sulfur binding center. Known [Fe-S]-binding residues in NarG of *E. coli* and the homologous positions in the other sequences are highlighted in yellow. The aspartate residue, which replaces one cysteine as a putative [Fe-S] ligand in the NxrA subunits of NOB02, Ca.N.defluvii, Ca. Nitrotoga fabula and Ca. Kuenenia stuttgartiensis is highlighted in gray. The conserved Molybdenum ligand binding site: aspartate residue was highlighted in wathet. (b): Known [Fe-S]-binding residues in NarH and the homologous positions in the NxrB sequences are highlighted in yellow. An aspartate residue, which replaces one cysteine as a putative [Fe-S] ligand in NxrB of NOB02, *Nitrospira* sp. CG24A, *N*. marine NB-295 and *Ca.* N. defluvii are highlighted in gary. An insertion, which is found only in the NxrB of NOB01, NOB02, *Nitrospira* sp. CG24A, *N*. marine NB-295 and *Ca.* N. defluvii, are highlighted in dark green **(c and d): phylogenetic analysis of *nxr*A and *nxr*B.** Tree was rooted by midpoint.


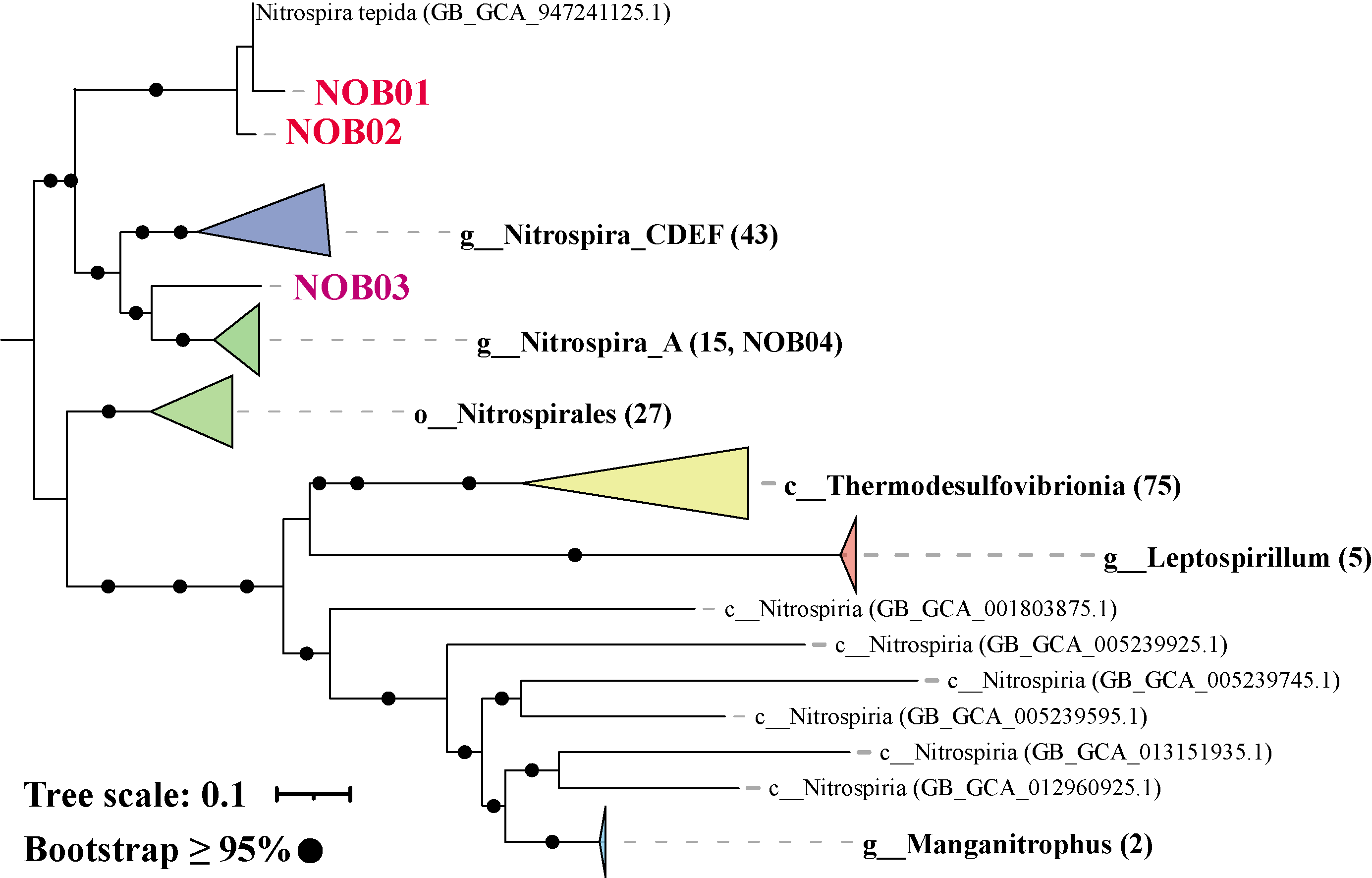


**Figure S4 GTDB-tk de_novo_wf analysis.** Tree was rooted by midpoint.


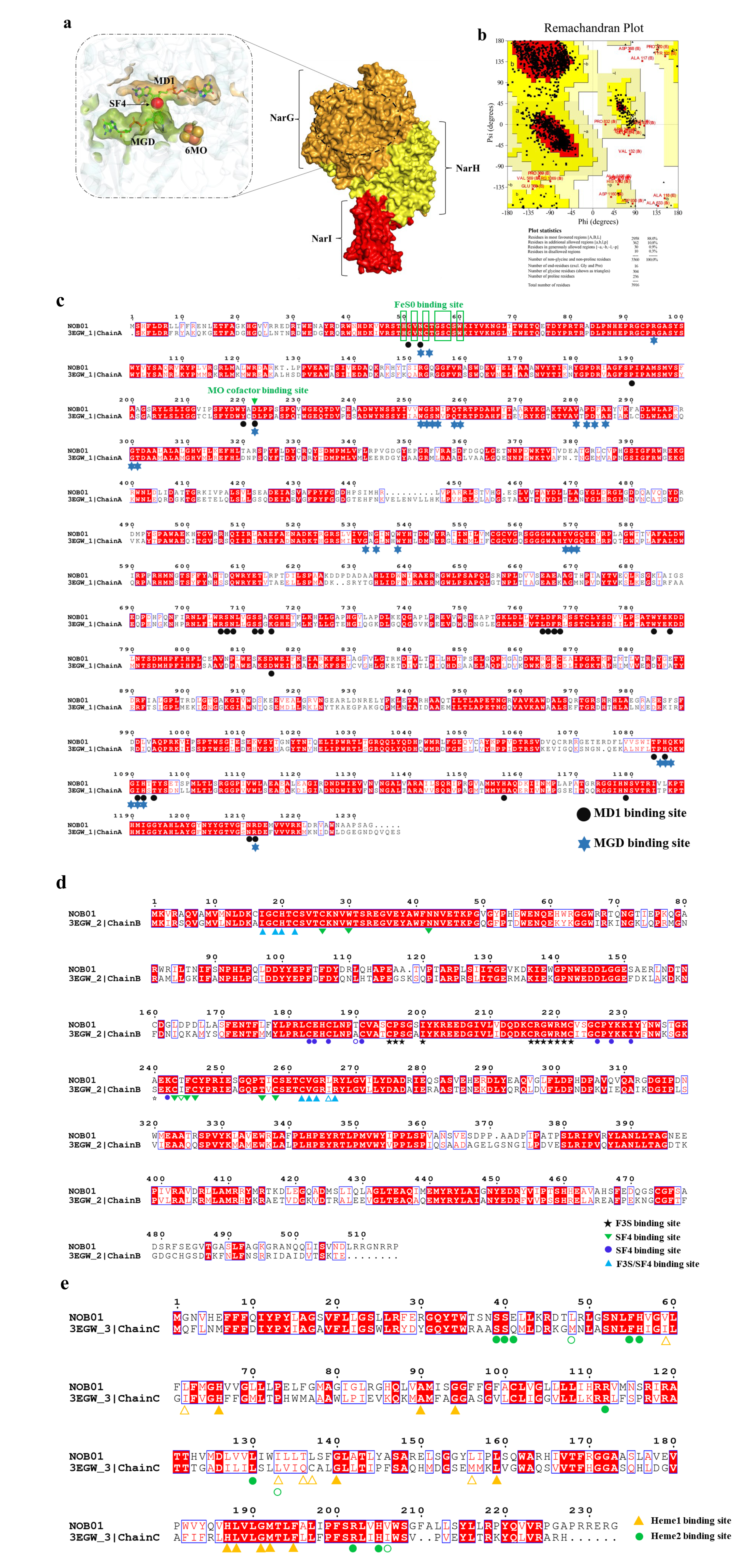


**Figure S5 (a):** **Evaluation of NAR model of NOB01, (c-e): Sequence alignment of NAR complexes with *E.coil* NAR (NarG: c, NarH: d, NarI: e) and zoom-in the ligand binding site.** The solid legends represent consistency, hollow legends represent inconsistency


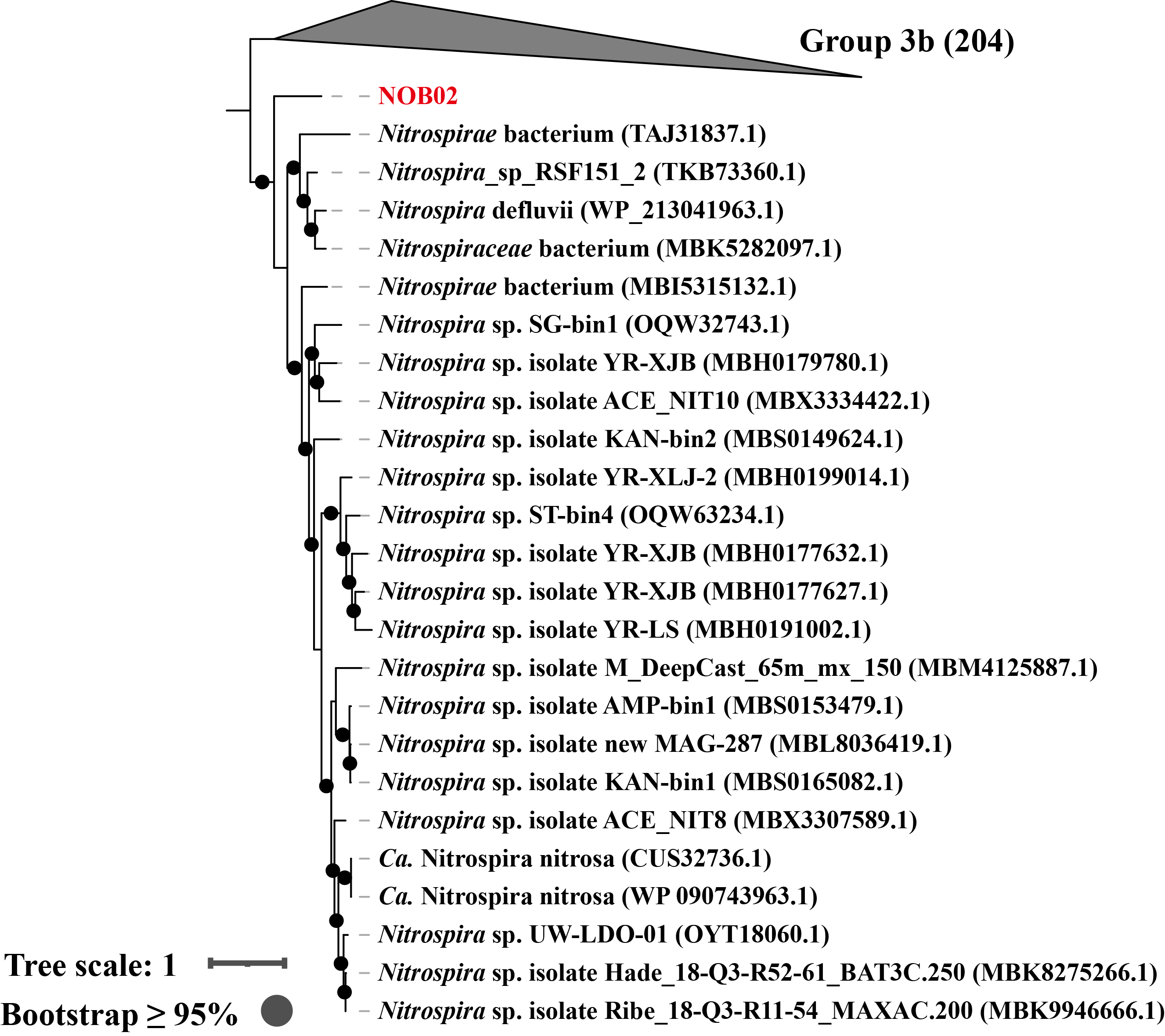


Figure S6 Phylogenetic analysis of Ni/Fe hydrogenase
